# The effects of a bacterial challenge on reproductive success of fruit flies evolved under low or high sexual selection

**DOI:** 10.1002/ece3.4450

**Published:** 2018-08-24

**Authors:** Magdalena Nystrand, Elizabeth J. Cassidy, Damian K. Dowling

**Affiliations:** ^1^ School of Biological Sciences Monash University Clayton Vic. Australia; ^2^ Department of Plant and Organismal Biology University of Copenhagen Copenhagen Denmark

**Keywords:** experimental evolution, life‐history, phenotypic plasticity, stress, trade‐offs

## Abstract

The capacity of individuals to cope with stress, for example, from pathogen exposure, might decrease with increasing levels of sexual selection, although it remains unclear which sex should be more sensitive. Here, we measured the ability of each sex to maintain high reproductive success following challenges with either heat‐killed bacteria or procedural control, across replicate populations of *Drosophila melanogaster* evolved under either high or low levels of sexual selection. Our experiment was run across four separate sampling blocks. We found an interaction between bacterial treatment, sexual selection treatment, and sampling block on female reproductive success. Specifically, and only in the fourth block, we observed that bacterial‐challenged females that had evolved under high sexual selection, exhibited lower reproductive success than bacterial‐challenged females that had evolved under low sexual selection. Furthermore, we could trace this block‐specific effect to a reduction in viscosity of the ovipositioning substrate in the fourth block, in which females laid around 50% more eggs than in previous blocks. In contrast, patterns of male reproductive success were consistent across blocks. Males that evolved under high sexual selection exhibited higher reproductive success than their low‐selection counterparts, regardless of whether they were subjected to a bacterial challenge or not. Our results are consistent with the prediction that heightened sexual selection will invoke male‐specific evolutionary increases in reproductive fitness. Furthermore, our findings suggest that females might pay fitness costs when exposed to high levels of sexual selection, but that these costs may lie cryptic, and only be revealed under certain environmental contexts.

## 1 INTRODUCTION

A central concept of life‐history theory is that resources are limited, and hence, life‐history traits must be traded off against each other (Roff, 2002). Natural selection is expected to act on these trade‐offs to optimize the performance of individuals in their local environments and facilitate adaptation to novel environments (Roff, 2002; Stearns, 1989). However, in most sexually reproducing species, evolutionary trajectories will also be influenced by sexual selection, prompted by females and males adopting different life‐history strategies to maximize their reproductive potential (Andersson, 1994).

Given that females generally produce few gametes of large size, while males produce numerous tiny sperm, life‐history theory predicts that females can increase their lifetime reproductive success by investing more into somatic maintenance and survival relative to males (Bateman, 1948; Parker, Baker, & Smith, 1972). Males, in contrast, will be expected to invest more heavily in traits that increase their reproductive vigor, but potentially at a cost to future survival and reproductive prospects (Andersson, 1994; Roff, 2002). Ultimately, this divergence in evolutionary interests between females and males should result in sex‐specific selection on optimal expression of life‐history phenotypes (Andersson, 1994; Bateman, 1948; Roff, 2002; Sisodia & Singh, 2004). Given the two sexes share the same genome (Pennell & Morrow, 2013), this can then precipitate trajectories of sexually antagonistic evolution by pulling each sex away from its phenotypic optima, both for the reproductive traits directly under sexual selection (Bonduriansky & Chenoweth, 2009), but in theory, also for a whole suite of correlated life‐history traits whose expression might trade‐off with these reproductive traits (Lessells, 2006; Parker, 1979).

One of the most effective ways to investigate the effects of sexual selection on population evolutionary trajectories is to experimentally modify the degree of sexual selection imposed on replicated populations across sequential generations and then compare phenotypic expression in fitness‐related traits across the different populations (Edward, Fricke, & Chapman, 2010; Kawecki et al., 2012; Singh & Singh, 2001). The organismal response to exposure to a pathogen is an example of a key fitness‐related trait, because it is expected to align positively with survival (Folstad & Karter, 1992; Lochmiller & Deerenberg, 2000; Zuk & Stoehr, 2002). This response is predicted to come at a cost and be traded off against investment in other life‐history traits and sexually selected traits (Dowling & Simmons, 2012; Folstad & Karter, 1992; Lochmiller & Deerenberg, 2000; McKean & Nunney, 2008; Zuk & Stoehr, 2002). Furthermore, organismal responses to pathogen exposure might also be expected to exhibit sex differences, given that the optimal resolution of the trade‐off between reproduction and survival should be specific to each sex, with females expected to invest more than males into their immune responses as a means of ensuring ongoing survival and hence augmenting their own lifetime reproductive success (McKean & Nunney, 2005; Nunn, Lindenfors, Pursall, & Rolff, 2009; Rolff, 2002; Zuk & Stoehr, 2002).

To date, a number of studies have tested for sex differences in various aspects of disease susceptibility, but the outcomes have not been consistent across studies (McKean & Nunney, 2005; Nunn et al., 2009; Short & Lazzaro, 2010; Winterhalter & Fedorka, 2009). However, only a handful of such studies have addressed these predictions within an experimental‐evolutionary framework, whereby different intensities of sexual selection were first applied to replicate populations, followed by some measurement of the immune capacity of the individuals of each population. Of the studies that have, some employed assays that measured components of “base‐level” immunity (i.e., when individuals were not first exposed to an immune challenge), for example, studies of yellow dung flies, *Scathophaga stercoraria* (Hosken, 2001), seed beetles, *Callosobruchus maculatus* (van Lieshout, McNamara, & Simmons, 2014), or meal moths, *Plodia interpunctella* (McNamara, Wedell, & Simmons, 2013). Others sought to gauge an individual's capacity to cope with an active pathogen challenge, for example, studies of fruit flies, *Drosophila melanogaster* (McKean & Nunney, 2008), and red flour beetles, *Tribolium castaneum* (Hangartner, Michalczyk, Gage, & Martin, 2015; Hangartner, Sbilordo, Michalczyk, Gage, & Martin, 2013). Furthermore, while these studies generally focused on estimating specific immunological parameters that are associated with innate immune responses (e.g., levels of enzyme activity [phenoloxidase] or antimicrobial activity [lysozyme‐like assays]), most of them also assayed some aspect of morphology or life‐history, such as body size or survival (Hangartner et al., 2013, 2015; Hosken, 2001; McKean & Nunney, 2008; van Lieshout et al., 2014). Yet, of all these studies, only one has so far detected clear sex differences in trait expression following an active pathogen challenge, in response to adaptation under sexual selection. In that study, conducted on red flour beetles, females from populations evolving under high, but not low, levels of sexual selection expressed higher baseline levels of the immune enzyme phenoloxidase than did males (Hangartner et al., 2015). With the caveat that there are generally too few studies yet with which to draw broad conclusions, collectively the current evidence suggests that adaptation under divergent regimes of sexual selection might not routinely lead to sex‐specificity in traits related to the immune response. But, clearly, further studies are required.

In this study, we test whether populations of the fruit fly *D. melanogaster*, which have been subjected to divergent levels of sexual selection, have evolved differences in the way in which they respond to exposure by a perceived pathogen, and whether any such responses vary between males and females. However, rather than studying the expression of *proximate* traits linked to immune system functionality, or morphological traits, as have most previous studies, here we instead focused on studying the expression of an *ultimate* and core life‐history trait—reproductive output—following exposure to either a bacterial challenge consisting of heat‐killed bacteria or a procedural control. Hence, our study focuses on changes in the expression of reproductive success (i.e., reproductive plasticity) in response to a noninfectious bacterial challenge, in populations that have evolved under divergent levels of sexual selection.

## 2 METHODS

### 2.1 Experimentally evolved populations

Experimentally evolved populations were originally obtained from Dr. Edward Morrow, University of Sussex, and their creation is described in detail in Innocenti, Flis, and Morrow (2014). In brief, 384 virgin female offspring and 384 virgin male offspring were collected from a well‐studied laboratory population (LH_M_) of flies, which was kept at large effective population size (Rice et al., 2006), and randomly allocated to one of eight replicate populations, which were then each assigned to a mating treatment of either low or high sexual selection.

Each replicate population was maintained over three 40 ml vials, each containing 16 pairs of adults, thus generating a population size of 96 flies per generation. Specifically, experimentally evolved populations were initially started by selecting 16 3‐day‐old virgin flies of each sex (16 pairs), which were placed into a yeasted (6 mg fresh yeast) vial to mate. In the low sexual selection treatment, flies were only provided with a total of 1 hr to mate and then separated. Extensive observations, aided by time‐lapse photography, show that under these conditions virtually all flies will mate once and once only (Innocenti et al., 2014; Kuijper & Morrow, 2009). Females in this treatment were then held within the same vials for 23 hr and then transferred to a fresh vial for 18‐hr period, in which they would lay the eggs that propagated the next generation. The number of eggs per vial was reduced to 150.

In the high sexual selection treatment, males were left in the vials to cohabit with the females for 24 hr. Flies were then transferred to fresh vials to enable them to lay eggs for 18 hr (these eggs propagated the next generation), after which the number of eggs per vial was reduced to 150. During this ovipositioning period, females from the high sexual selection treatment were accompanied by the males with whom they cohabited. Previous data on these lines have confirmed that females start to remate after approximately 2 hr when kept under *en masse* conditions (Innocenti et al., 2014). Moreover, studies in other *D. melanogaster* populations have demonstrated remating rates of 80%–100% in young females (3–4 days old) within the 20‐hr mark (Fricke, Green, Mills, & Chapman, 2013), and remating rates of 30%–50% within 6 hr under multiple pair conditions (van Vianen & Bijlsma, 1993). Ten days later, the eclosing virgin flies of each of the three vials per population replicate were admixed and then resorted into sex‐specific vials (three vials of 16 flies per sex), such that every population was kept across three vials each generation. Experimental populations were kept under standard rearing conditions of 25°C, 12 L:12D, and 60% humidity.

We received a copy of each of these experimental populations in 2011, at which point they had already evolved for 95 generations. All experimental populations were maintained under an identical schedule in our laboratory for another 80 generations, apart from three modifications. First, we increased the number of vials per replicate population to seven, and thus, each population was represented by 224 flies per generation. Second, the medium substrate used once the populations arrived in our laboratory was based on a potato‐dextrose‐agar medium (37.32% yeast, 31.91% dextrose, 23.40% potato, and 7.45% agar combined with 98.48% H_2_O, 0.97% ethanol, 0.45% propionic acid, and 0.11% nipagen), rather than the cornmeal‐molasses‐yeast‐agar medium that was used up until 2011. Third, the number of eggs per vial was trimmed to 120. The populations evolved for 80 generations on this new medium prior to the start of our experiments.

Note, that while Innocenti et al. (2014) previously called these treatments “Monogamous (M)” and “Promiscuous (P),” we refer to them as low (L) and high (H) sexual selection treatments, as these classifications more accurately describe the conditions under which each population evolved. Promiscuity implies mating indiscriminately at random, whereas female remating patterns in *D. melanogaster* are known to shape by nonrandom factors (Byrne & Rice, 2006; Dickson, 2008; Dukas, 2005; Edward & Chapman, 2012; Filice & Long, 2017). Experimental evolution studies that manipulate mating systems (monogamy vs. polyandry/polygyny/promiscuity) do so in order to test the effects of adaptation under low and high levels of sexual selection. Populations that evolve under monogamy have low opportunities/magnitudes of sexual selection. While precopulatory sexual selection can operate under such conditions, postcopulatory bouts of sexual selection are eliminated (since females only house the sperm of one male within their reproductive tracts). Populations that evolve under polyandry/polygyny/promiscuity have higher opportunities/magnitudes of sexual selection. First, in these populations, females are exposed to males for longer (42 hr) than they are exposed to males in the monogamous setting (1 hr), and this provides more opportunity for precopulatory processes of sexual selection. Females exhibit a strong physiological refractory period between matings that can last up to 24 hr (Brown, Bjork, Schneider, & Pitnick, 2004; Manning, 1962, 1967), but which can sometimes be much shorter (Fricke et al., 2013; Innocenti et al., 2014; van Vianen & Bijlsma, 1993). Males will vigorously court females during this time, and thus, levels and durations of precopulatory behaviors are higher than under the monogamous setting, and this gives females ample opportunity to able to “trade‐up” by remating with other males. Second, in populations that evolve under polyandry/polygyny/promiscuity, females remate with other males, and this puts the sperm of two males in direct competition within the females’ reproductive tract, thus invoking postcopulatory sexual selection (Simmons, 2001). Third, in populations that evolve under polyandry/polygyny/promiscuity, sexually antagonistic selection, a form of sexual selection, becomes relevant, given a clear conflict over the optimal mating rate in these systems (females need to mate just one to secure the sperm that will fertilize their clutch, while males increase their fitness as a function of the number of females that they mate with) (Arnqvist & Rowe, 2005; Bateman, 1948).

In the experiments described below, we use a full copy of eight of the replicated experimental evolution populations described above. All populations were maintained for one generation under common garden conditions, prior to the start of the experiment, to reduce the potential for nongenetic parental effects to affect the results of the study. To generate the focal flies in our experiment (i.e., those that were the focus of our experimental treatments), we used a crossing scheme in which we mated females of each replicate population of a given selection regime to males of a different replicate population from the same selection regime. This scheme is outlined in Table 1. Although there is no evidence that our replicate populations have suffered from reductions in effective population size, leading to effects of inbreeding depression, which could in theory be more prevalent in one‐or‐the‐other of the selection treatment, the effect of this crossing scheme is to eliminate the potential that our interpretations are confounded by effects of selection regime‐specific inbreeding effects. The focal flies of each selection treatment type will carry a full haploid genome inherited from one given replicate population, and a full haploid genome inherited from another population that has evolved under the same selection treatment. Under this design, effects of additive, dominant, recessive, or epistatic variation that has evolved consistently under each selection treatment, across each of the four replicate populations per treatment, will be detectable. Furthermore, additive, dominance, and epistatic variation that has evolved specifically in one or other of the replicate populations (i.e. *not* consistently across each population) will also be detectable and can be modeled by inclusion of the replicate population cross‐combination, in the statistical models (see Section 2.5 below). However, we note that recessive variants that have evolved specifically in one or other replicate populations, to the sexual selection treatment, will be undetectable, given that these variants would likely be masked by dominant counterparts contributed from the other replicate population used in the crossing scheme. While, in theory, this is a limitation of our design, in practice recessive variation is thought to contribute little to the dynamics of adaptation, simply because these variants will remain masked from selection unless they are at high frequencies in populations (so‐called Haldane's sieve; (Charlesworth, 1992; Haldane, 1924, 1927; Turner, 1981)—a scenario that would presumably only occur following a large population bottleneck. Such population bottlenecks have not occurred in our replicate populations, which have been maintained at high effective population sizes as their inception to minimize the effects of drift in shaping these populations (Haldane, 1924; Olson‐Manning, Wagner, & Mitchell‐Olds, 2012).

**Table 1:** Parental crossing scheme (L = low sexual selection, H = high sexual selection; number “1–4” denotes the four population replicates for each selection treatment)

Crosses used to create focal flies		Focal fly × Tester fly matings	Crosses used to create tester flies	
Female	Male		Female	Male
L1	L4	L1L4 × L4L1	L4	L1
L2	L3	L2L3 × L3L2	L3	L2
L3	L2	L3L2 × L2L3	L2	L3
L4	L1	L4L1 × L1L4	L1	L4
H1	H4	H1H4 × H4H1	H4	H1
H2	H3	H2H3 × H3H2	H3	H2
H3	H2	H3H2 × H2H3	H2	H3
H4	H1	H4H1 × H1H4	H1	H4

*Note*

The focal flies were produced by crossing flies from two separate replicate populations within the same selection treatment (e.g., L1 females to L4 males, with this particular cross then denoted L1L4). These cross‐combinations are depicted in the left‐hand column. The tester flies were produced in the same way, but using the reciprocal cross (e.g., L4L1), and these are depicted in the right‐hand column. In the subsequent assays of reproductive success, focal flies of each cross‐combination were then mated to the tester flies produced by the reciprocal cross (these matings are depicted in the central column).

The “tester” flies that were used to mate to the focal flies in the experiment were collected from the same crossing design (Table 1). The focal flies of one sex were always mated to tester flies of the other sex that had also evolved under the same selection treatment, but produced from the reciprocal population cross to that which produced the focal flies (e.g., focal flies of cross L1L4 in Table 1 were mated to tester flies of cross L4L1). This ensured that none of the offspring produced during the assays of reproductive success were the result of consanguineous matings (Table 1).

### 2.2 Bacterial treatment

A total of 435 females and 415 males were collected as virgins from the focal low sexual and high sexual selection population crosses, and flies of each sex were then stored across two separate vials, per cross‐combination (*X*_females_ [*SE*] = 6.80 ± 0.16; *X*_males_ [*SE*] = 6.48 ± 0.18 flies per vial). Three days later (i.e., as 3‐day‐old adult flies), individuals from each vial were randomly allocated into two groups, with one group then subjected to a noninfectious bacterial challenge (i.e., recognized by the immune system but representing a nonreplicating pathogen) and the other to a procedural control (Supporting Information Table S1).

Both the bacterial challenge and the control were administered via microinjection (using “Nanoject,” Drummond Scientific Company, Broomall, PA, USA) into the abdomen, at a volume of 41.1 nl per fly. We utilized a bacterial challenge that was noninfectious, by ensuring the bacteria used were heat‐killed. Hence, the bacterial challenge consisted of a mix of heat‐killed Gram‐negative bacteria (*Escherichia coli,* strain K12, OD600 = 1.0, corresponding to ~27.5 × 10^6^ CFU per fly) and heat‐killed Gram‐positive bacteria (*Micrococcus luteus strain,* A204, OD600 = 0.1, corresponding to ~1.1 × 10^6 ^CFU per fly), which was diluted in phosphate‐buffered saline (PBS; Sigma‐Aldrich table P4417, pH 7.4). The bacterial batch was made up once.

Both heat‐killed and live *E. coli* and *M. luteus* have been used in previous studies to successfully induce an immune response in *D. melanogaster* (Lemaitre & Hoffmann, 2007; Nehme et al., 2011; Zerofsky, Harel, Silverman, & Tatar, 2005), and both bacteria, when heat‐killed, have been previously shown to affect the expression of components of reproduction on the test subjects or their offspring, following a bacterial challenge (Nystrand, Cassidy, & Dowling, 2016, 2017; Nystrand & Dowling, 2014b; Zerofsky et al., 2005). The advantage of using heat‐killed bacteria in this assay was that it enabled us to specifically explore the costs of the expression of reproductive success induced by the host physiological response to the challenge per se, without the associated effects of disease that are induced when infected by replicating, living pathogens (Nystrand & Dowling, 2014a, 2014b; Nystrand et al., 2016). Moreover, the bacterial concentrations adopted in this study were informed by previous experiments in our laboratory that explored the effects of different heat‐killed bacteria, and different doses of each, on host reproductive success, relative to a procedural‐ and a naïve‐control (Nystrand & Dowling, 2014b). The heat‐killed bacteria were sourced and verified to be heat‐killed by colony growth test, from *Micromon Genomics, Biotechnology, and Diagnostics* facility (Monash University, Australia).

### 2.3 Reproductive assays

Twenty‐four hours following the microinjections, the 4‐day‐old focal flies were placed into fresh vials, individually, with a 7‐day‐old “tester” fly of the opposite sex. Tester flies were sampled from the same selection treatment as the focal flies, but resulted from the reciprocal population cross (Table 1). In the assay of female reproductive success, the focal and tester flies were kept together for 24 hr to enable mating, after which the females were transferred to a fresh vial containing a new tester male. This procedure was repeated over four consecutive days, keeping the tester male age constant at 7 days of age (achieved by collecting newly eclosed tester males over a period of four consecutive days), after which the female was discarded. In a previous study on this species, we demonstrated that the total number of offspring produced per female over the first 4 days of ovipositioning correlated strongly with the total number generated across the first 10 days (during which time the majority of eggs have been deposited), and thus, this assay should provide a sufficient proxy of female reproductive success (Nystrand & Dowling, 2014b).

**Figure 1:**
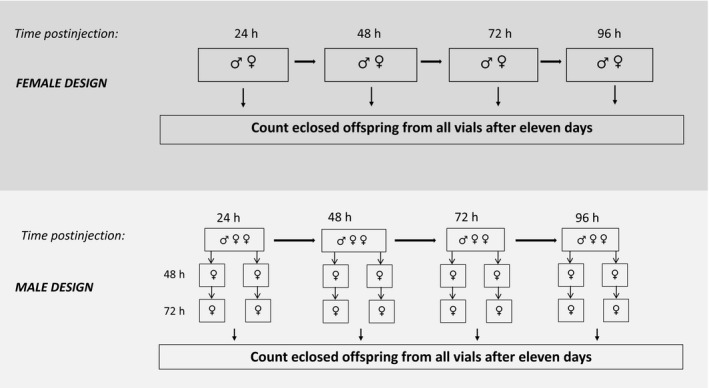
Outline of experimental design for both sexes, where each of the square boxes denoted on the figure (each of which contains male [♂] and/or female [♀] symbols) symbolizes a vial. In the female assay, each focal female was transferred to new ovipositing vial every 24 hr, over 96 hr, and each of these vials contained a virgin tester male whom was removed after 24 hr. In the male assay, each focal male was placed in a vial with two virgin tester females for 24 hr, after which he was transferred to a new vial containing two new virgin females for 24 hr, and this process was repeated every 24 hr over a 96‐hr period. After each 24‐hr period, the two tester females were transferred to their own individual vials at 24‐hr intervals over the next 48 hr (i.e., 48 hr and 72 hr postmating). Each vial was retained for 11 days, at which point eclosing offspring were counted

Male reproductive success is less constrained by the number of gametes that a male can produce in a given time and thus increases as a function of the number of females a male has access to (Bateman, 1948). In the assay of male reproductive success, we therefore provided each focal male with a total of eight females over the course of a 4‐day‐long assay. During the first 24 hr of mating, each experimental male, at 4 days of age, was placed with two 7‐day‐old virgin tester females. Following 24 hr of cohabitation, the tester females were each transferred to their own individual vials and then transferred to a second vial 24 hr later, to enable ovipositioning across two vials per female. The experimental males, however, were transferred to a fresh vial containing another two 7‐day‐old tester virgin females, and this process continued over 4 days (such that each male had the opportunity to mate with eight females in total). In sum, the reproductive data for females were based on four vials, whereas that for males were based on a total of 20 vials (see Figure 1 for a schedule on experimental outline).

All mating vials consisted of potato‐dextrose‐yeast medium, but only vials used prior to the mating (prior to the reproductive success assay) contained ad libitum live yeast applied to the surface of the medium. Eleven days after each 24 hr ovipositioning period (all flies had eclosed at this stage), the total number of eclosed offspring was counted per vial. The total number of offspring eclosing per focal female and focal male over these assays was then used as our measures of female and male reproductive success.

### 2.4 Temporal outline of experiment

The experiment was conducted across four sampling blocks, each separated by one generation. All four blocks were executed in the same, controlled laboratory environment. However, in the first three blocks, females oviposited on, and offspring of the focal flies were reared on, a high‐viscosity potato‐dextrose‐agar medium, which is used as per standard in our laboratory. In the fourth block, however, supply issues forced us to use a different batch of agar (provided by the same supplier, Gelita Australia, Agar‐Agar). We observed that this medium had markedly lower viscosity, but it was otherwise identical in its nutritional composition to the standard medium used in the laboratory. Indeed, the altered viscosity of the agar in this block had clear effects on the reproductive success of both the focal females, and the tester females in the male assay, as illustrated by the mean number of offspring produced per female per medium (focal females, *X*_block1–3_ = 139 ± 2 *SE*,* N* = 328, and *X*_block4_ = 212 ± 5 *SE*,* N* = 107; tester females, presenting means averaged across the eight tester females that each male mated with, *X*_blocks 1–3_ = 82 ± 1 *SE*,* N* = 311, and *X*_block4_ = 115 ± 3 *SE*,* N* = 104). In sum, in the fourth block, females laid many more eggs per vial than in previous blocks.

### 2.5 Statistical analysis

All statistical analyses were run in R v. 3.1.1 (R Development Core Team, 2012), using the glmmADMB package (Skaug, Fournier, Nielsen, Magnusson, & Bolker, 2012). Both male and female data were substantially overdispersed when fitted with a Poisson error distribution and conformed better to the negative binomial error distribution (fitted with a NB1, which has a variance = *ϕμ*). The dependent variable for the male and female models was the number of eclosed offspring, and explanatory variables were the “selection treatment” (low vs. high sexual selection), “bacterial treatment” (bacterial challenge vs. control), block (block 1–4; each separated in time by one generation), and all possible interactions between these effects. Random factors were “vial identity” (shared vial environment before transfer to individual vials), identity of the selected population cross that generated the focal flies (construction of L1–L4 and H1–H4 parental crosses, *n* = 8 levels, Table 1), and an additional grouping variable accounting for the dependence between crosses that shared part of their genome (four levels, e.g., L1L4 and L4L1 = Parental Cross Group 1, L2L3 and L3L2 = Group 2, H1H4 and H4H1 = Group 3, and H2H3 and H3H2 = Group 4). Statistical models were assessed using log‐likelihood ratio tests, utilizing the drop1() function in R, to compare the change in deviance associated with the deletion of each term from the model. Estimation of significance was based on chi‐square tests of deviances, and an α‐criterion of 0.05. Full models are displayed, with the most parsimonious (final) model based on AIC values associated with each model (i.e., lowest AIC) denoted in bold in Table 2.

**Table 2:** Effect of *bacterial treatment* (bacterial challenge or control), *selection treatment* (low sexual selection or high sexual selection), and *experimental block* on (a) female and (b) male reproductive success. Note that the fourth (4) block consisted of a slightly altered environment compared to the other blocks (i.e., lower viscosity of the food medium). Log‐likelihood ratios (LRT) and the associated *p*‐values were generated from log‐likelihood tests between nested models whereby the full model was compared to a reduced model (single term deletion). The most parsimonious model (lowest AIC) is in bold writing

Fixed factors	LRT	Pr > Chi‐sq
(a)		
Selection treatment	**0.02**	**0.8875**
Bacterial treatment	**6.62**	**0.0101**
Block	**86.90**	<**0.001**
Bacterial treatment × selection treatment	**0.02**	**0.8875**
Bacterial treatment × block	**5.36**	**0.1473**
Selection treatment × block	**8.12**	**0.0436**
Bacterial treatment × selection treatment × block	**12.04**	**0.0072**
*Random effects*	*Full model variance*	
Vial identity	**0.0044**	
Parental crossing scheme	<**0.001**	
Parental crossing scheme group	**0.0072**	
(b)		
Selection treatment	**6.86**	**0.0088**
Bacterial treatment	**0.68**	**0.4096**
Block	**33.16**	<**0.001**
Bacterial treatment × selection treatment	0.02	0.8875
Bacterial treatment × block	2.98	0.3947
Selection treatment × block	3.74	0.2909
Bacterial treatment × selection treatment × block	0.22	0.9743
*Random effects*	*Full model variance*	
Vial identity	**0.0135**	
Parental crossing scheme	**0.0017**	
Parental crossing scheme group	**1.13** ^**−07**^	

The results for each sex were analyzed in separate models, because the reproductive traits measured in females and males were not directly comparable and were acquired from different assays.

## 3 RESULTS

**Figure 2:**
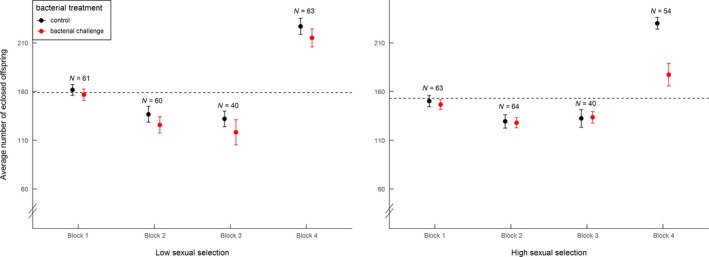
Effect of *bacterial treatment* (bacterial challenge or control) and *selection treatment* (low sexual selection or high sexual selection) on female reproductive success, shown across the four different experimental blocks. Graphs show raw data (mean ± *SE*), with total sample sizes per block displayed above the means. Average mean reproductive success is (across blocks and treatment) is illustrated by a horizontal hatched line

There was no general effect of the sexual selection treatment on female reproductive success. However, an interaction between the selection treatment, bacterial treatment, and block affected the reproductive success of females (Table 2a). This effect was attributable to the bacterial challenge only inducing a negative effect on female reproductive success in the high sexual selection treatment and then only in the fourth experimental block (Figure 2). We further investigated whether this block‐specific pattern was attributable to some of the population replicates behaving as outliers. However, within both selection treatments, each population replicate exhibited a near‐identical response to the bacterial treatment within this block (Supporting Information Figure S1, i–iv). This uniformity across replicates provides strong evidence that the responses in Block 4 were driven by consistent differences in the test environment that were particular to this block (i.e., lower viscosity of the medium), and which had led to females laying more eggs per vial (see Section 2 and Supporting information Figure S2).

**Figure 3:**
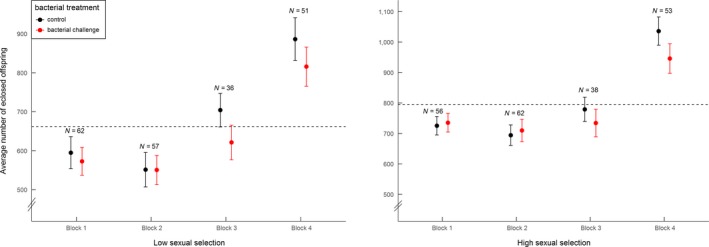
Effect of *bacterial treatment* (bacterial challenge or control) and *selection treatment* (low sexual selection or high sexual selection) on male reproductive success, shown across the four different blocks. Graphs show raw data (mean ± *SE*), with total sample sizes per block displayed above the means. Average mean reproductive success (across blocks and treatment) is illustrated by horizontal hatched line

In contrast, male reproductive performance was largely unaffected by the bacterial treatment, regardless of block (Table 2b, Figure 3). However, males from populations evolving under high sexual selection exhibited a generally higher reproductive success than their counterparts from populations evolving under low sexual selection (Table 2b, Figure 3, also see Additional file for main effect, Supporting information Figure S3). Similar to females, males also had a generally higher reproductive success in Block 4, attributable to the tester females laying more eggs (Figure 3, Supporting information Figure S4).

## 4 DISCUSSION

Adaptation under high levels of sexual selection is predicted to carry costs, particularly when it comes to optimal expression of traits that enhance somatic maintenance and survival phenotypes (Andersson, 1994; Bateman, 1948; Roff, 2002). In particular, high levels of sexual selection are likely to increase the reproductive output of males, but potentially at the expense of investment into somatic maintenance (Bonduriansky & Brassil, 2005; Maklakov, Bonduriansky, & Brooks, 2009). Given a strong genetic correlation between the sexes for most traits (Bonduriansky & Chenoweth, 2009), this might have negative consequences for females, by dragging this sex away from its optimal expression of traits involved in reproductive fitness and traits involved in somatic maintenance and survival (Arnqvist & Rowe, 2005). Thus, strong sexual selection for traits involved in sexual interactions may lead to negatively correlated responses of other traits that are key to survival. Currently, however, it is not clear whether such correlated responses commonly occur, when it comes to the capacity to cope with exposure to perceived or real pathogens; and moreover, whether such responses are common across both sexes, or limited to one or other of the sexes.

The primary goal of our study was, therefore, to determine whether reproductive outputs of male and female fruit flies were modified when challenged with a noninfectious bacteria (heat‐killed bacteria), and whether any such responses were in turn affected by the history of sexual selection on the sampled populations. Our results were striking, and we discuss them below.

First, we confirmed a general effect of the sexual selection treatment on male reproductive success, with males from populations evolving under high levels of sexual selection exhibiting a generally higher reproductive success. Such a response to adaptation under sexual selection has been commonly observed in experimental evolution studies that have applied sexual selection on replicated populations of other invertebrate species, for example, in the fruit fly, *D*. *pseudoobscura* (Crudgington, Fellows, Badcock, & Snook, 2009), the red flour beetle, *T*. *castaneum* (Michalczyk et al., 2011), the dung beetle, *Onthophagus taurus* (Simmons & Garcia‐Gonzalez, 2008), and the yellow dung fly *S*. *stercoraria* (Hosken, Garner, & Ward, 2001). Indeed, this result is opposite in direction to that of a previous study in *D. melanogaster,* in which males from populations that had evolved under relaxed sexual selection (“monogamy”) had higher reproductive success following a single mating event to a standardized female, compared to flies that had evolved under competitive conditions (Pitnick, Miller, Reagan, & Holland, 2001). Note, however, in that study, when flies were mated under competitive conditions, this relationship was reversed. The main difference between that early study and our study is that males in our experiment were mated to co‐evolved females rather than standardized females, thereby allowing evolved counter‐adaptation to male‐induced harm to be expressed in the interacting females. Hence, the higher reproductive success in high sexual selection males was concordant with expectations.

While we did not formally investigate the underpinning mechanisms that led to males having higher reproductive performance under high sexual selection, previous studies have suggested that heightened sexual selection typically leads to the evolution of larger testis size and/or spermatocyte production, as indicated in previous studies utilizing experimental evolution approaches in *D*. *melanogaster* (Pitnick et al., 2001), and yellow dung flies (*Scatophaga stercoraria*) (Hosken & Ward, 2001; Hosken et al., 2001), and from comparative studies of frogs (Byrne, Roberts, & Simmons, 2002) and primates (Harcourt, Purvis, & Liles, 1995). Notwithstanding this evidence, other studies that have altered levels of sexual conflict and sexual selection in *D. melanogaster* have actually failed to find differences in basic testes size or accessory gland size (Chechi, Ali Syed, & Prasad, 2017; Linklater, Wertheim, Wigby, & Chapman, 2007; Wigby & Chapman, 2004). Rather, the evidence from *Drosophila* suggests that any changes in male reproductive outcomes following evolution under divergent regimes of sexual selection may be explained by increased rates of accessory gland protein (ACP) depletion in populations evolving under high sexual selection, during repeated matings (possibly to give them a competitive advantage under real or perceived competition) (Linklater et al., 2007), or changes to the actual *composition* of seminal fluid proteins (e.g., ACPs) (Wigby et al., 2009). Alternatively, the increased reproductive performance of high sexual selection males may be more closely related to changes in precopulatory behaviors (which indeed may be triggered by physiological changes (Wigby et al., 2009)), as have been shown in a study on *D*. *melanogaster*, in which populations evolving under high sexual selection evolved higher male courtship rates in concert with elevated female remating rates (Holland & Rice, 1999).

Notably, no general augmenting effect of high sexual selection on female reproductive success was observed in our study, and nor was there an interaction between the bacterial treatment and the sexual selection treatment on reproductive success in the first three experimental blocks, during which time the flies were measured in their standard nutritional environments. However, we did observe a complex interaction between the bacterial treatment, sexual selection treatment, and the experimental block on reproductive output. Specifically, reproductive outputs were lower for females that had been subjected to the bacterial treatment, but only for females sampled from high sexual selection populations, and only in the fourth block. This intriguing pattern suggests that females evolving in populations subjected to high levels of sexual selection may suffer a genetic cost that their “low sexual selection” counterparts do not experience, when it comes to their capacity to maintain high reproductive output following bacterial exposure, but that this cost is context‐dependent and may only manifest itself under certain environmental conditions. In particular, the fourth block of this experiment was noticeably different than the preceding three, and this was demonstrated by significantly increased egg yields per vial in this block (around 50% higher than those of the previous three blocks) that were presumably precipitated by the lower viscosity of the laying substrate of the vials of this block. While we refrain from drawing any firm conclusions about the processes underlying this block‐specific result, we suggest that more work be devoted to further exploring the role that environmental heterogeneity could play in moderating genetic costs of adaptation under sexual selection.

It is worth addressing why males did not suffer reductions in reproductive success following bacterial exposure, particularly males of populations that evolved under high sexual selection. A general prediction, informed by life‐history theory, is that males should invest less into somatic maintenance than females, as high reproductive output in males can be optimized by mating with many females early in life, at the expense of survival (Andersson, 1994; Roff, 2002). As such, it follows that males would show less phenotypic plasticity in reproductive output following a pathogen exposure, as under this predicted trade‐off, males would gain most by continuing to invest heavily in early‐life reproduction, regardless of risks to longer term survival, and regardless of the environmental context. Notwithstanding this, we acknowledge that other studies have previously tested for plasticity in traits tied to male reproductive success, following exposure to nonpathogenic immune elicitors. Many of these have focused on components of sperm quality, and they have generally recorded negative effects of a pathogen exposure; for example, in studies of fruit flies, *D. melanogaster* (Radhakrishnan & Fedorka, 2012), moths, *Heliothis armigera* (McNamara, van Lieshout, Jones, & Simmons, 2013), house crickets, *Gryllodes sigillatus* (Kerr, Gershman, & Sakaluk, 2010), and field crickets, *Telegryllus oceanicus* (Simmons, 2012). Other studies have explored the effects of noninfectious challenges on male mate calling patterns in crickets, and these have also observed effects linked to exposure. For example, ground crickets (*Allonemobius socius*) had larger interpulse song intervals when exposed to *S. marcescens*‐derived LPS (Fedorka & Mousseau, 2007), field crickets (*Gryllus campestris*) showed an initial decrease in calling rate when exposed (Jacot, Scheuber, & Brinkhof, 2004), and Texas field crickets (*Gryllus texensis*) displayed a context‐dependent increase in calling effort when LPS‐challenged (Kelly, Telemeco, & Bartholomay, 2015). Interestingly, however, and consistent with our results, studies that have examined traits that are more directly aligned with the outcomes of male reproduction per se, such as patterns of mating success in moths, *Heliothis virescens* (Barthel, Staudacher, Schmaltz, Heckel, & Groot, 2015) and crickets, *A. socius* (Fedorka & Mousseau, 2007), or offspring production in *D. melanogaster* (Nystrand & Dowling, 2014a; Nystrand et al., 2016, 2017), have not found effects of pathogen exposure on trait expression.

Finally, it is possible that we would have seen stronger effects overall, had we used a live, replicating bacteria to challenge hosts. While the mechanistic pathways of the immune system in insects (*Drosophila* in particular) are relatively well understood (Lemaitre & Hoffmann, 2007), less is known about what aspects of an immune stress ultimately drive immune‐mediated life‐history trade‐offs. In theory, the organismal response could be caused primarily by the pathological effects associated the activities of a particular pathogen inducing physiological costs to the host (e.g., manipulation of host resources, interference with signaling pathways and the cellular environment, behavioral manipulation) (Agudelo‐Romero, Carbonell, Perez‐Amador, & Elena, 2008; Fedorka & Mousseau, 2007; Grindstaff, Hunsaker, & Cox, 2012; Lochmiller & Deerenberg, 2000; Paschos & Allday, 2010; Sadd & Siva‐Jothy, 2006). Alternatively, the primary costs of an immune stress could be caused by the direct effects of the host redistributing resources away from reproduction and survival to fighting disease (Lochmiller & Deerenberg, 2000; Roff, 2002; Sheldon & Verhulst, 1996). By administering heat‐killed bacteria, we effectively factored out a large component of the direct costs associated with disease, and honed in on the specific costs associated with the deployment of the immune system. This is, however, likely to have reduced the overall impact of infection on mediating life‐history trade‐offs involving reproductive outcomes.

In conclusion, we uncovered a general effect of sexual selection on male reproductive performance. In females, we recorded a context‐dependent and block‐specific effect of bacterial challenge on reproductive output, across populations of fruit flies with divergent histories of sexual selection. These results pose an important question; To what degree are the costs associated with evolution under sexual selection moderated by context dependence such as environmental conditions? Sex‐specificity in the degree to which costs of sexual selection are manifested across environments could have an underrated influence on shaping trajectories of trait evolution (Ingleby, Hunt, & Hosken, 2010). Natural populations are constantly exposed to heterogeneous and changing environments (Anderson, Wagner, Rushworth, Prasad, & Mitchell‐Olds, 2014; Candolin & Heuschele, 2008), and there are plenty of examples to support widespread gene by environmental effects in a range of phenotypic traits (Bashir‐Tanoli & Tinsley, 2014; Fanara, Folguera, Iriarte, Mensch, & Hasson, 2006; Howick & Lazzaro, 2014; Lazzaro, Flores, Lorigan, & Yourth, 2008; Nystrand, Dowling, & Simmons, 2011). Thus, there is a clear need for more research exploring evolutionary trajectories of populations under divergent levels of sexual interaction across a range of environmental contexts. The outcomes could provide new insights into the evolutionary costs and benefits that accrue to each sex in populations evolving under heightened sexual selection.

## CONFLICT OF INTEREST

None declared.

## AUTHOR'S CONTRIBUTION

MN designed and coordinated the study, conducted statistical analyses, and wrote the manuscript; EC carried out laboratory work and contributed to design; and DKD helped to design the study and write the manuscript.

## AVAILABILITY OF DATA AND MATERIALS

The dataset supporting the conclusions of this article will be available in Figshare upon manuscript acceptance.
