# Supplementary material for: The effects of a bacterial challenge on reproductive success of fruit flies evolved under low or high sexual selection
Source: Ecol Evol. 2018 Aug 24;8(18):9341–52. doi: 10.1002/ece3.4450 (PMC6194216; doi:10.1002/ece3.4450)
Supplement: Supplementary file 1 [file ECE3-8-9341-s001.pdf]

THE EFFECTS OF A BACTERIAL CHALLENGE ON REPRODUCTIVE SUCCESS OF FRUIT FLIES EVOLVED UNDER LOW OR HIGH SEXUAL SELECTION

Nystrand, M*, Cassidy, EJ., & Dowling, DK.

TABLE OF CONTENTS

A) ADDITIONAL METHODS

I. Table S1. Average number of flies per vial in each treatment.

II. Additional information on the bacterial challenge.

B) ADDITIONAL FIGURES

Figure S1. Effect of bacterial treatment on female reproductive success across each selected population replicate (from focal populations L1L4, L2L3, L3L2, L4L1 and H1H4, H2H3, H3H2, H4H1; see Table 1 in main manuscript for more details) in block four (4).

Figure S2. Effect of *bacterial treatment* (bacterial challenge or control) and *selection treatment* (low *vs.* high sexual selection) on female reproductive success, across block 1-3 *vs*. block 4.

Figure S3. Main effect of sexual selection on male reproductive success (raw means ± SE).

Figure S4. Main effect of block on male reproductive success (raw means ± SE).

C) REFERENCES

Additional Methods:

Table S1. Average number of flies per vial in each treatment.

		Bacterial challenge		Control challenge	
	Nvials	X number /vial	SE	X number/vial	SE
**Females**	64	3.44	0.09	3.36	0.10
**Males**	64	3.23	0.11	3.25	0.10

Additional information on the bacterial challenge:

We have previously used challenges with heat-killed bacteria – either *M. luteus* in isolation [1], or a mix of *M. luteus* and *E. coli* [2, 3], as well as with the immune-elicitor lipopolysaccharide from *Serratia marcescens* [4], to show effects on the expression of reproductive success. Hence, we are confident that a challenge with these bacteria has the capacity to invoke a phenotypic response in the flies.

In our previous experiments, when comparing effects of the heat-killed bacterial treatment to controls, reproductive success of the test subjects responded similarly to both the naïve control (no handling treatment) and the procedural control consisting of an injection of PBS solution [1, 3]. Therefore, we only included the procedural control in the current experiment, to be able to fully disentangle the effect of the heat-killed bacteria from that of injury alone. Hence, the flies of the procedural control received only the PBS, administered by the same method and at the same volume as the bacteria-challenged flies. Injected flies were then transferred to fresh vials, none of which contained live yeast.

Additional Tables and Figures:

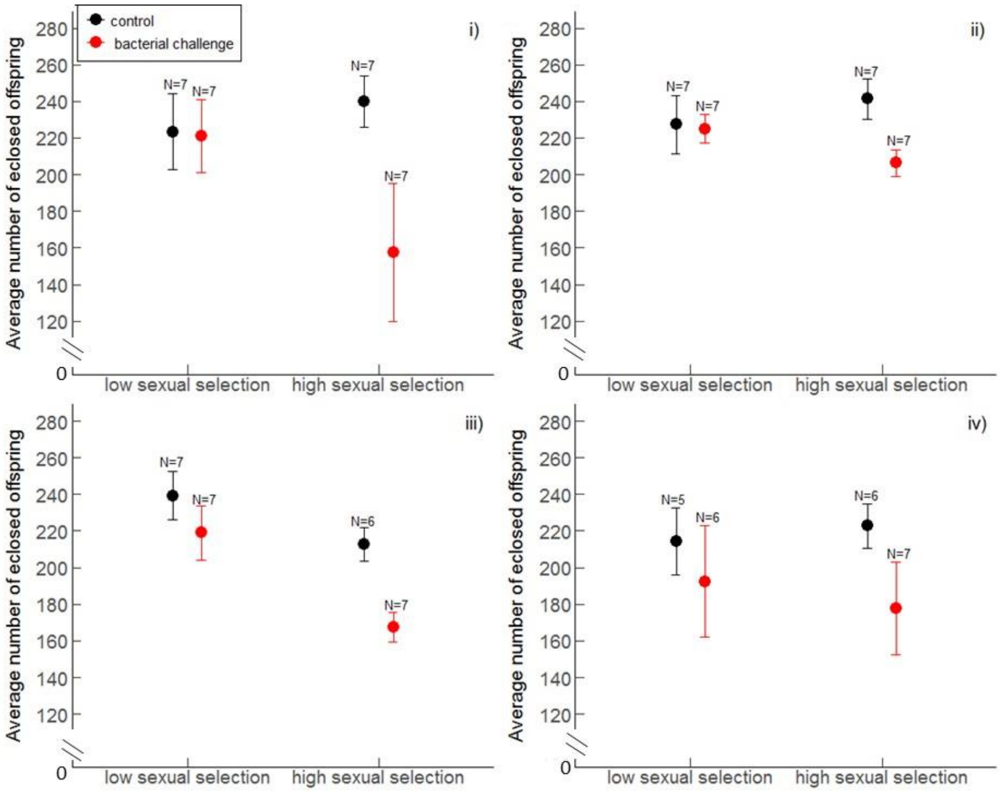

Figure S1. Effect of bacterial treatment on female reproductive success across each population replicate (L1L4, L2L3, L3L2, L4L1 and H1H4, H2H3, H3H2, H4H1; where panel (i) shows L1L4 and H1H4, panel (ii) shows L2L3 and H2H3 etc.), in block four (4).

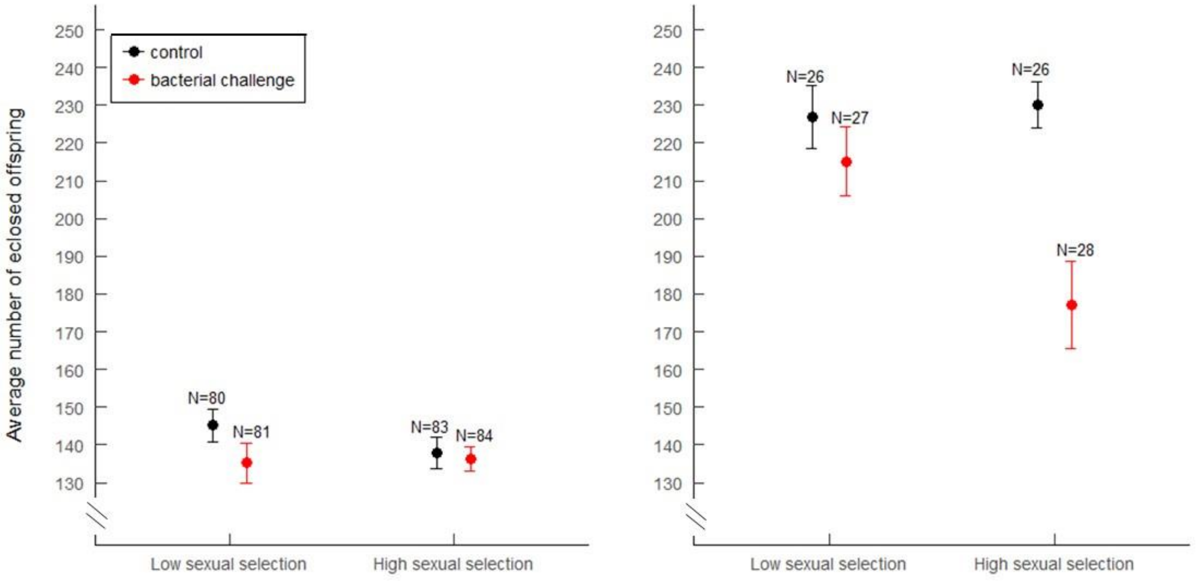

Figure S2. Effect of *bacterial treatment* (bacterial challenge or procedural control) and *selection treatment* (low sexual selection or high sexual selection) on female reproductive success, in block 1-3 (left panel) *versus* block4 ("low-viscosity medium"), right panel. Graphs are based on raw data (mean ± SE, with sample sizes displayed across each selection treatment group.

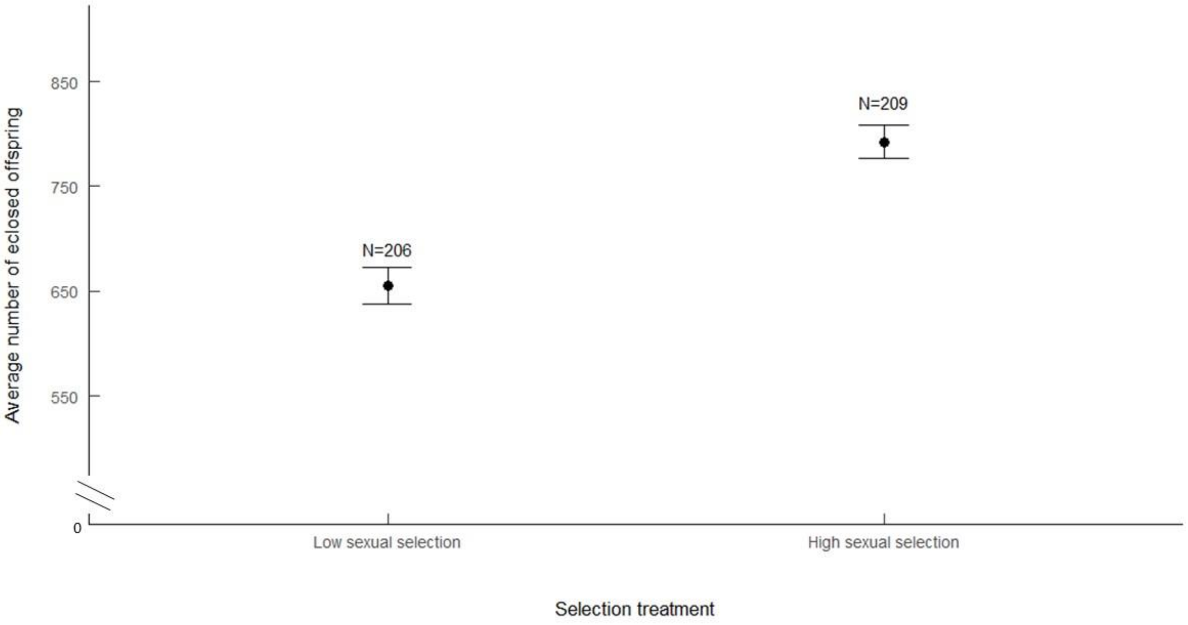

Figure S3. Main effect of sexual selection on male reproductive success (raw means ± SE).

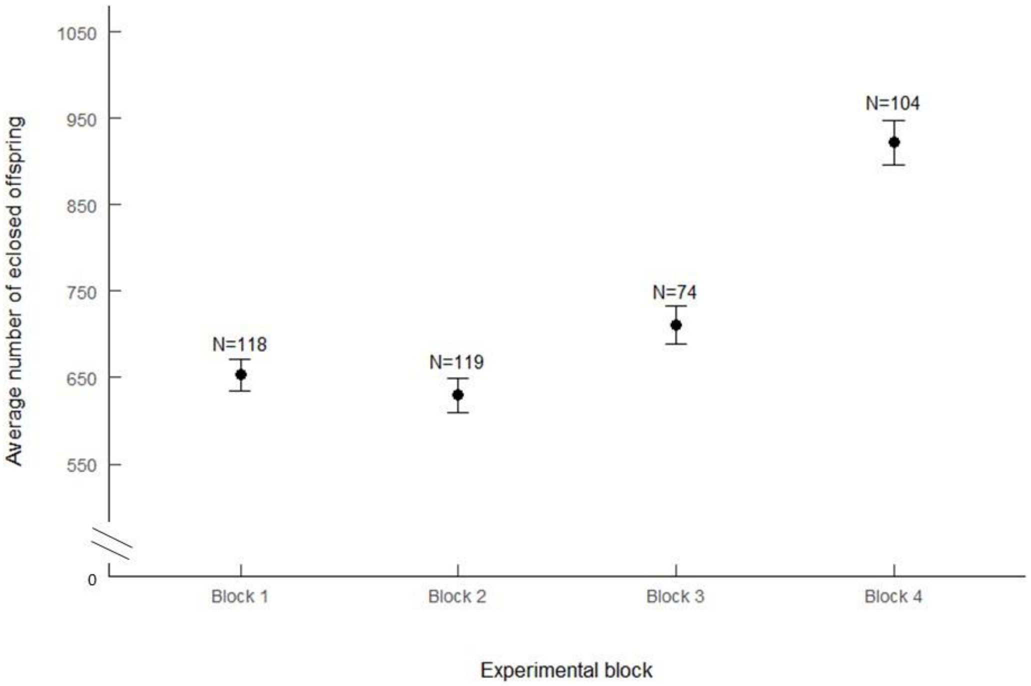

Figure S4. Main effect of block on male reproductive success (raw means ± SE).

REFERENCES

[1] Nystrand, M. & Dowling, D.K. 2014 Transgenerational interactions involving parental age and immune status affect female reproductive success in Drosophila melanogaster. *Proc. R. Soc. Lond., Ser. B: Biol. Sci.* **281**. (doi:10.1098/rspb.2014.1242).

[2] Nystrand, M., Cassidy, E.J. & Dowling, D.K. 2017 No effect of mitochondrial genotype on reproductive plasticity following exposure to a non-infectious pathogen challenge in female or male Drosophila. *Sci. Rep.* **7**, 42009. (doi:10.1038/srep42009. http://www.nature.com/articles/srep42009#supplementary-information).

[3] Nystrand, M., Cassidy, E.J. & Dowling, D.K. 2016 Transgenerational plasticity following a dual pathogen and stress challenge in fruit flies. *BMC Evol. Biol.* **16**, 1-11. (doi:10.1186/s12862-016-0737-6).

[4] Nystrand, M. & Dowling, D.K. 2014 Dose-dependent effects of an immune challenge at both ultimate and proximate levels in Drosophila melanogaster. *J. Evol. Biol.*, 876-888. (doi:10.1111/jeb.12364).
